# Automated 3D MRI rendering of the craniofacial skeleton: using ZTE to drive the segmentation of black bone and FIESTA-C images

**DOI:** 10.1007/s00234-020-02508-7

**Published:** 2020-08-08

**Authors:** Karen A Eley, Gaspar Delso

**Affiliations:** Department of Radiology, University of Cambridge School of Clinical Medicine, Box 218, Cambridge Biomedical Campus, Hills Road, Cambridge, CB2 0QQ UK

**Keywords:** Skull, Three-dimensional imaging, Magnetic resonance imaging, Facial bones, Image processing, computer-assisted]

## Abstract

**Purpose:**

Automated bone segmentation from MRI datasets would have a profound impact on clinical utility, particularly in the craniofacial skeleton where complex anatomy is coupled with radiosensitive organs. Techniques such as gradient echo black bone (GRE-BB) and short echo time (UTE, ZTE) have shown potential in this quest. The objectives of this study were to ascertain (1) whether the high-contrast of zero echo time (ZTE) could drive segmentation of high-resolution GRE-BB data to enhance 3D-output and (2) if these techniques could be extrapolated to ZTE driven segmentation of a routinely used non bone-specific sequence (FIESTA-C).

**Methods:**

Eleven adult volunteers underwent 3T MRI examination with sequential acquisition of ZTE, GRE-BB and FIESTA-C imaging. Craniofacial bone segmentation was performed using a fully automated segmentation algorithm. Segmentation was completed individually for GRE-BB and a modified version of the algorithm was subsequently implemented, wherein the bone mask yielded by ZTE segmentation was used to initialise segmentation of GRE-BB. The techniques were subsequently applied to FIESTA-C datasets. The resulting 3D reconstructions were evaluated for areas of unexpected bony defects and discrepancies.

**Results:**

The automated segmentation algorithm yielded acceptable 3D outputs for all GRE-BB datasets. These were enhanced with the modified algorithm using ZTE as a driver, with improvements in areas of air/bone interface and dense muscular attachments. Comparable results were obtained with ZTE+FIESTA-C.

**Conclusion:**

Automated 3D segmentation of the craniofacial skeleton is enhanced through the incorporation of a modified segmentation algorithm utilising ZTE. These techniques are transferrable to FIESTA-C imaging which offers reduced acquisition time and therefore improved clinical utility.

## Introduction

The widespread use of three-dimensional (3D) imaging and 3D printing has further fuelled the search for automated segmentation methods, with resultant high clinical expectations irrespective of intrinsic imaging limitations. The unrivalled benefits of soft tissue imaging of MRI combined with a desire to avoid ionising radiation exposure have led to a quest for rapid bone imaging techniques which can obviate the need for CT imaging.

The potential benefits of automated 3D reconstructed MRI are perhaps no greater than in the craniofacial skeleton, where complex anatomy is coupled with radiosensitive organs (lens and thyroid gland) and often benign pathology in young patients. The challenge in segmentation of the skull lies in the fact that compact bone is characterised by very short T2* relaxation times and relatively short T1 relaxation times [1]. In an attempt to circumnavigate the difficulty in obtaining signal from bone, gradient echo (GRE) black bone (BB) techniques were developed to enhance the bone-soft tissue boundary using a combination of short echo (TE) and repetition times (TR) and a low flip angle. This results in minimal signal being returned from cortical bone and soft tissue species which therefore appear uniform. The main benefit of this GRE-based technique is that it can be implemented on all MRI systems irrespective of vendor or field strength. Previous attempts with GRE-BB to produce 3D rendered imaging of the craniofacial skeleton have shown considerable promise, but remain limited by time-intensive segmentation techniques [2–8]. Recent developments in an automated segmentation algorithm have brought these techniques significantly closer to routine clinical practice [9].

Ultrashort echo time (UTE) and more recently zero echo time (ZTE) imaging have been increasingly used to evaluate structures with the shortest T2 values, with demonstrated benefits in PET/MRI attenuation correction, with demonstrable segmentation success within the skull [1, 10]. Unlike the conventional RF pulse sequences, the ZTE sequence begins a readout period immediately after the RF pulse, thus permitting visualisation of short T2 materials [11].

A potential synergy between BB and ZTE arises from the different nature of their intrinsic limitations: in the case of BB, fully automated segmentation methods are complicated due to the uncertainty between air and bone tissue voxels, both yielding no detectable signal. Conversely, short echo time methods, like ZTE, are capable of detecting the signal from bone tissue and thus discerning it from air. However, these methods are characterised by a radial k-space acquisition pattern (which is not time efficient), as well as low flip angles, requiring multiple averages to achieve sufficient signal-to-noise ratio for segmentation purposes. In consequence, the image resolutions that can be effectively achieved by ZTE in clinical practice are currently limited to ~ 1 mm^3^.

The primary objective of this study was to ascertain whether the 3D imaging output of BB could be enhanced by using the high contrast of ZTE to drive segmentation of the high-resolution imaging of GRE-BB data. The secondary objective was to ascertain if this segmentation approach could be applied to a routine MRI sequence, which is not optimised for bone imaging. FIESTA-C (fast imaging employing steady-state acquisition; GE) and the equivalent CISS (constructive interference steady state; Siemens) sequences are volume acquisition sequences used in a wide range of imaging protocols providing high-resolution T2-weighted GRE imaging. Manual segmentation of the skull on FIESTA-C imaging has recently been demonstrated to be feasible [12]. Yielding data suitable for 3D reconstruction from routinely acquired sequences would provide clear clinical advantages in terms of MRI examination efficiency.

## Materials and methods

Following ethical approval (Cambridge Human Biology Research Ethics Committee, HBREC.2016.13), 11 adult volunteers underwent MRI examination on a 3T system (SIGNA PET/MR, GE Healthcare, MP26 software version). The age range of volunteers was between 28 and 39 years, with a male predominance (female *n* = 4; male *n* = 7).

The protocol included the sequential acquisition of ZTE (3D radial), GRE-BB and FIESTA-C imaging. The imaging parameters are shown in Table 1. All imaging was completed using a 21-element head and neck receive coil (GE GEM HNU).

**Table 1:** Imaging acquisition parameters

	BB	FIESTA-C	ZTE
TR [ms]	7.3	5.7	456.9
TE [ms]	2.1	2.1	0.0
FA [deg]	5.0	17.0	0.8
BW [Hz/Px]	128	355	488
NEX	2	1	4
Matrix [Px^3^]	512x512x212	512x512x424	256x256x150
Spacing [mm^3^]	0.5 × 0.5 × 1.2	0.4 × 0.4 × 0.7	0.9 × 0.9 × 1.6

**Fig. 1:**
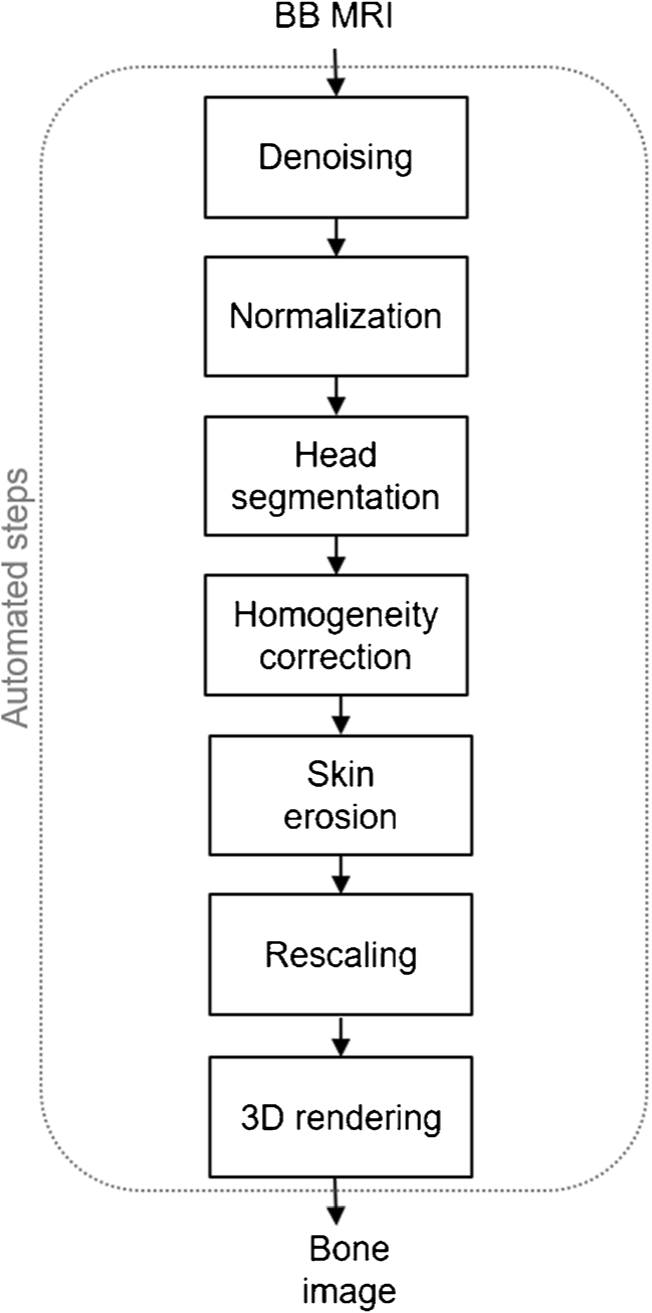
Block diagram of the automated bone rendering algorithm, relying only on black bone images (BB-GRE)

**Fig. 2:**
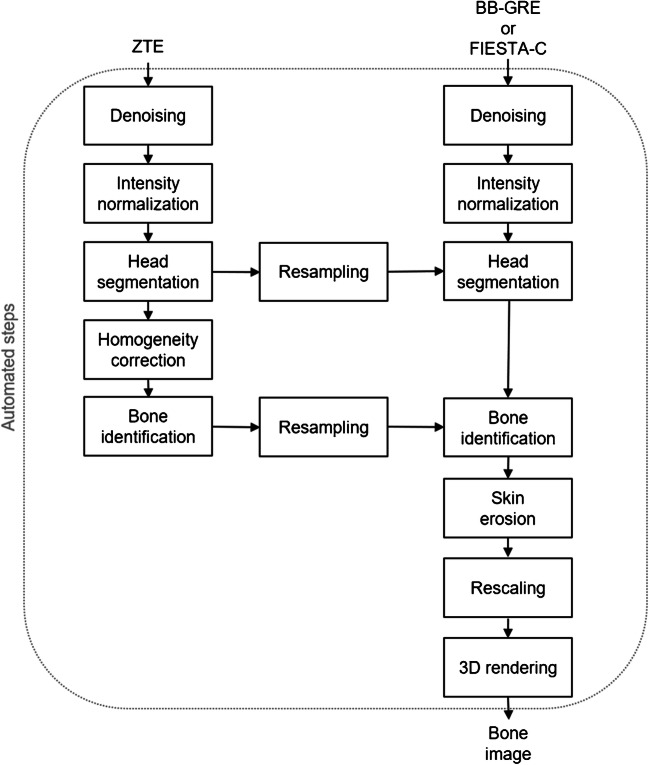
Block diagram of the new bone rendering algorithm, relying on low-resolution ZTE data to enhance the depiction of high-resolution data (BB-GRE or FIESTA-C)

Craniofacial bone segmentation was performed using a fully automated segmentation algorithm, implemented in C++ using the Insight Segmentation and Registration Toolkit (ITK), including image denoising, intensity normalisation, head mask generation, N4 bias correction, skin removal, intensity rescaling and masking (Fig. 1). Segmentation was firstly completed for BB. A modified version of the algorithm was then implemented (Fig. 2), wherein the bone mask yielded by ZTE segmentation was used to initialise the segmentation of BB (ZTE+BB). The two segmentation algorithms were subsequently applied to the FIESTA-C datasets.

An automated 3D rendered image with rotating movie clip was produced for each of the datasets.

Since acquisition of comparative CT imaging data would be unethical in this volunteer setting, assessment of the 3D reconstructed imaging was completed in terms of the expected findings with unexpected discrepancies such as bone defects being compared with the raw datasets using Osirix (Osirix MD, Version 10.0.5, Pixmeo SARL, Switzerland). Fine manipulation of the 3D rendered imaging was also performed using Osirix.

## Results

**Fig. 3:**
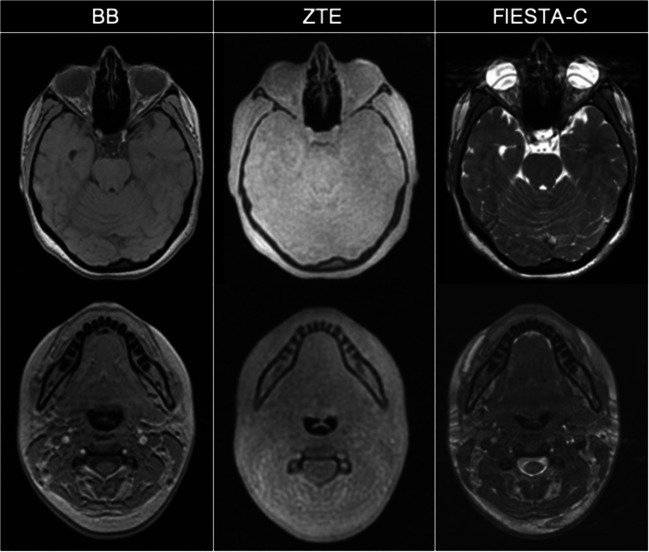
Axial acquisition of BB, ZTE and FIESTA-C demonstrating the inherent low-resolution imaging of ZTE when compared with BB and FIESTA-C

The acquisition times for the three sequences were 1:16, 4:48 and 3:28 min for ZTE, BB and FIESTA-C, respectively. Examples of the three axial acquisitions are shown in Fig. 3. The automated segmentation algorithm was successfully applied to all of the BB datasets.

**Fig. 4:**
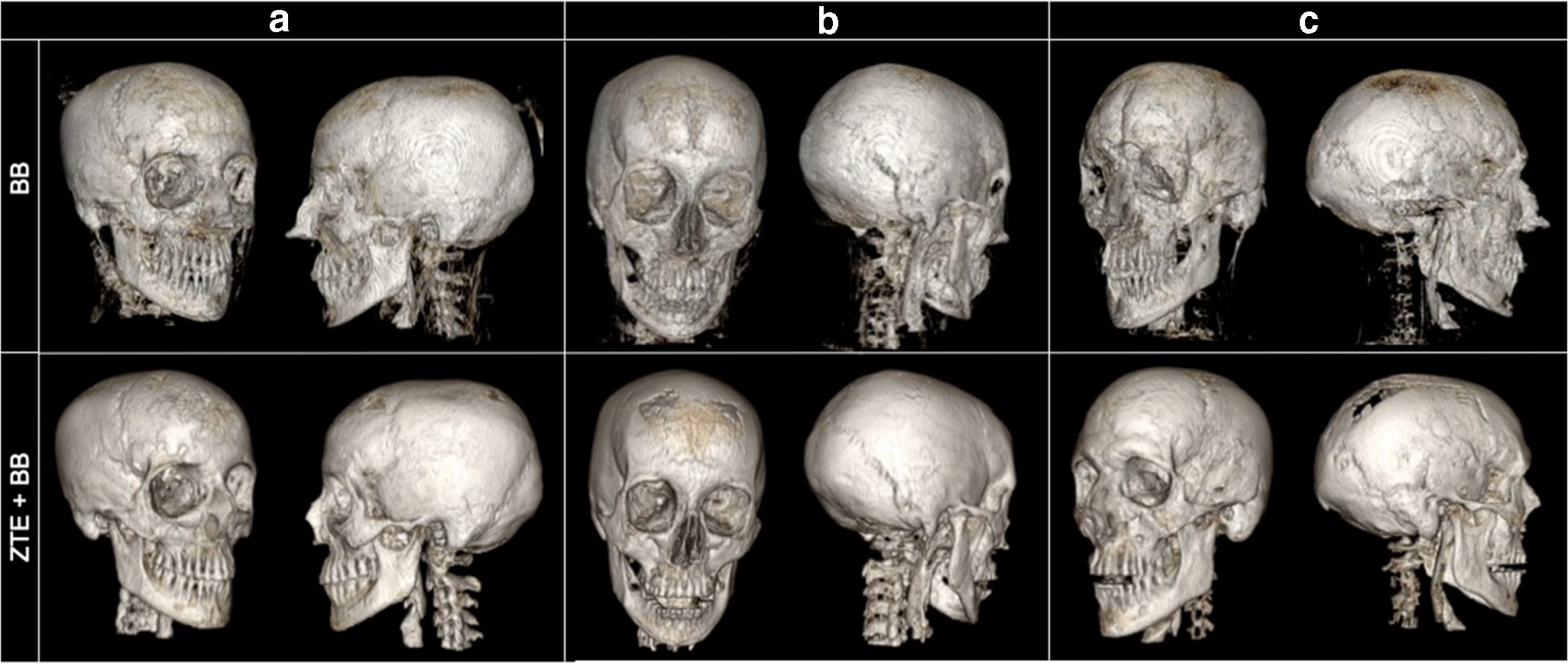
Automated 3D reconstruction of the craniofacial skeleton in adult volunteers to highlight differences between BB and BB+ZTE segmentation algorithms. Note the improved accuracy of BB+ZTE in terms of removal of cartilaginous and muscular structures. Areas of discrepancy are noted overlying the frontal sinus (**a**), and lateral orbital wall (**b**) and skull vertex (**c**) on BB images, with some improvement seen on the modified BB+ZTE results

**Fig. 5:**
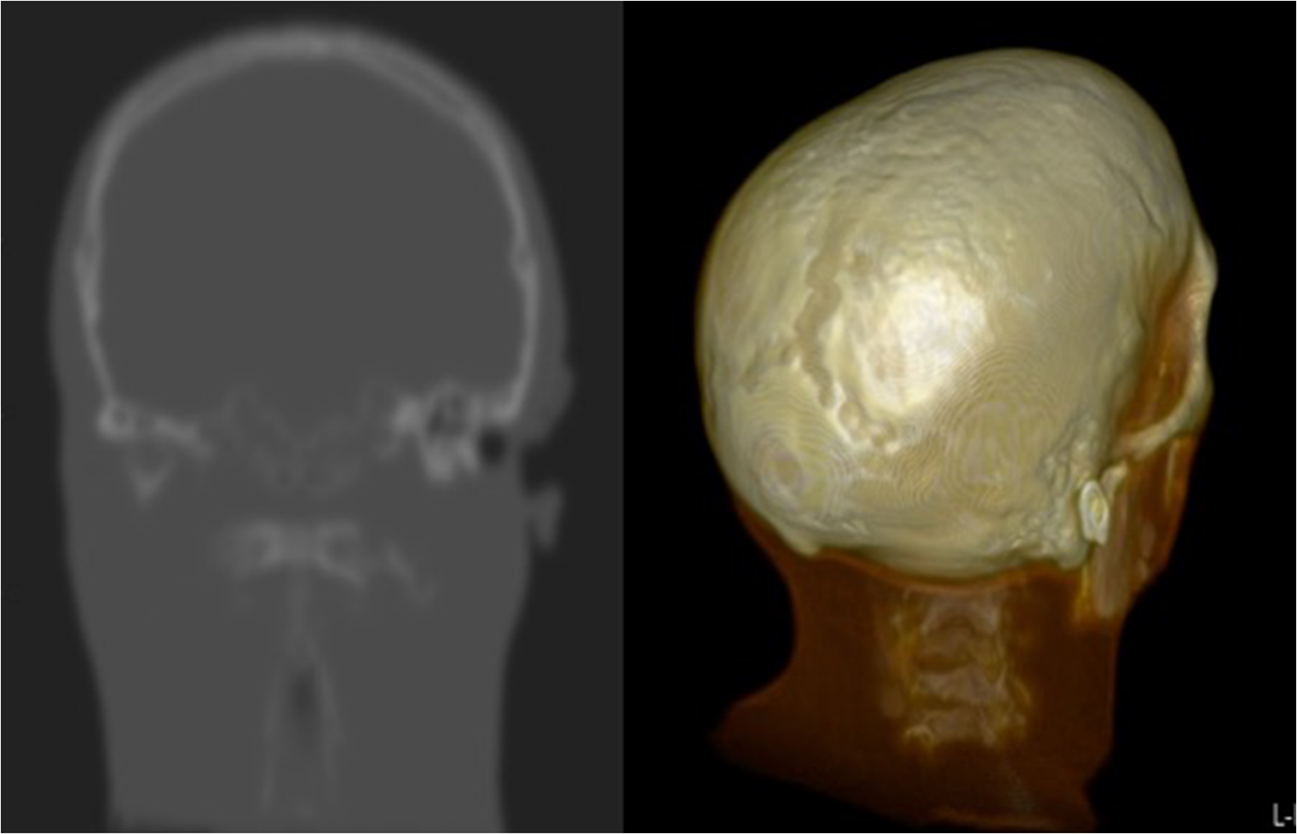
Post-processed raw BB coronal imaging and 3D volume rendering of the dataset shown in Fig. 4C. This demonstrates the absence of bony defects at the skull vertex, highlighting that these defects are most likely a post-processing phenomenon and thus resolvable with future refinement of the technique

**Fig. 6:**
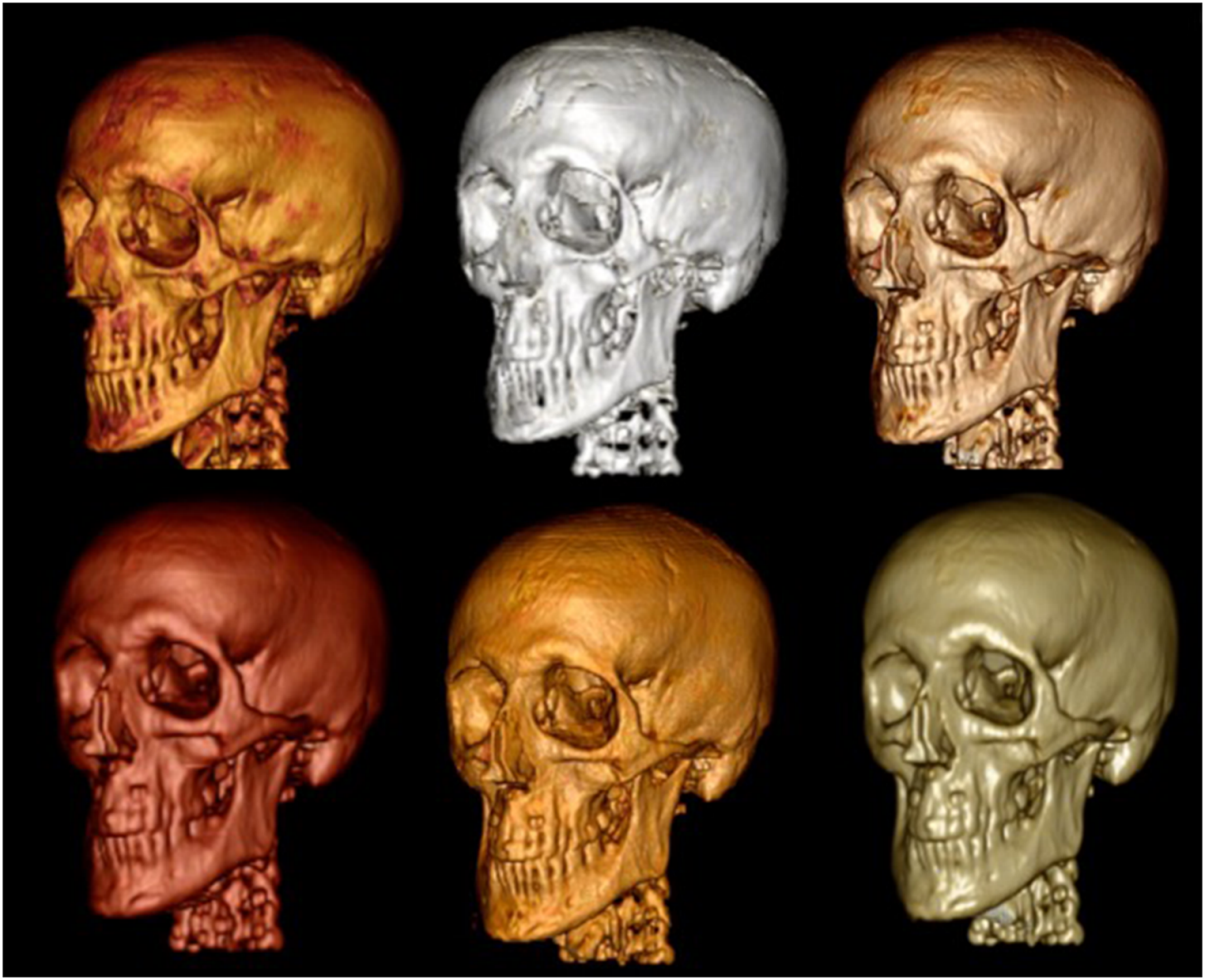
Fine manipulation of the final 3D-rendered imaging is possible by the end-user utilising software such as Osirix. Shown here are a range of 3D imaging from the same processed BB-ZTE dataset

The combined acquisition time for BB+ZTE was 6:04 min. The 3D renderings were enhanced with the modified algorithm utilising ZTE to drive the BB segmentation (Fig. 4) with the modified algorithm successful in removing non-bony tissues such as ligaments and muscles, which were often incompletely achieved in the initial segmentation method with BB alone. Other areas of discrepancy in the initial algorithm were also seen in a small number of cases in regions where the bone was particularly thin, such as the orbit, which were also resolved with the modified algorithm in the majority of cases. Full and partial thickness bone defects were occasionally encountered at the skull vertex, the skull base and overlying the frontal sinus, on the 3D BB and BB+ZTE automated 3D renderings. These discrepancies were not always present on the source datasets nor the processed raw datasets, and could therefore be resolved using Osirix (Fig. 5). Further manipulation of the final 3D results was possible using Osirix (Fig. 6) to optimise output.

**Fig. 7:**
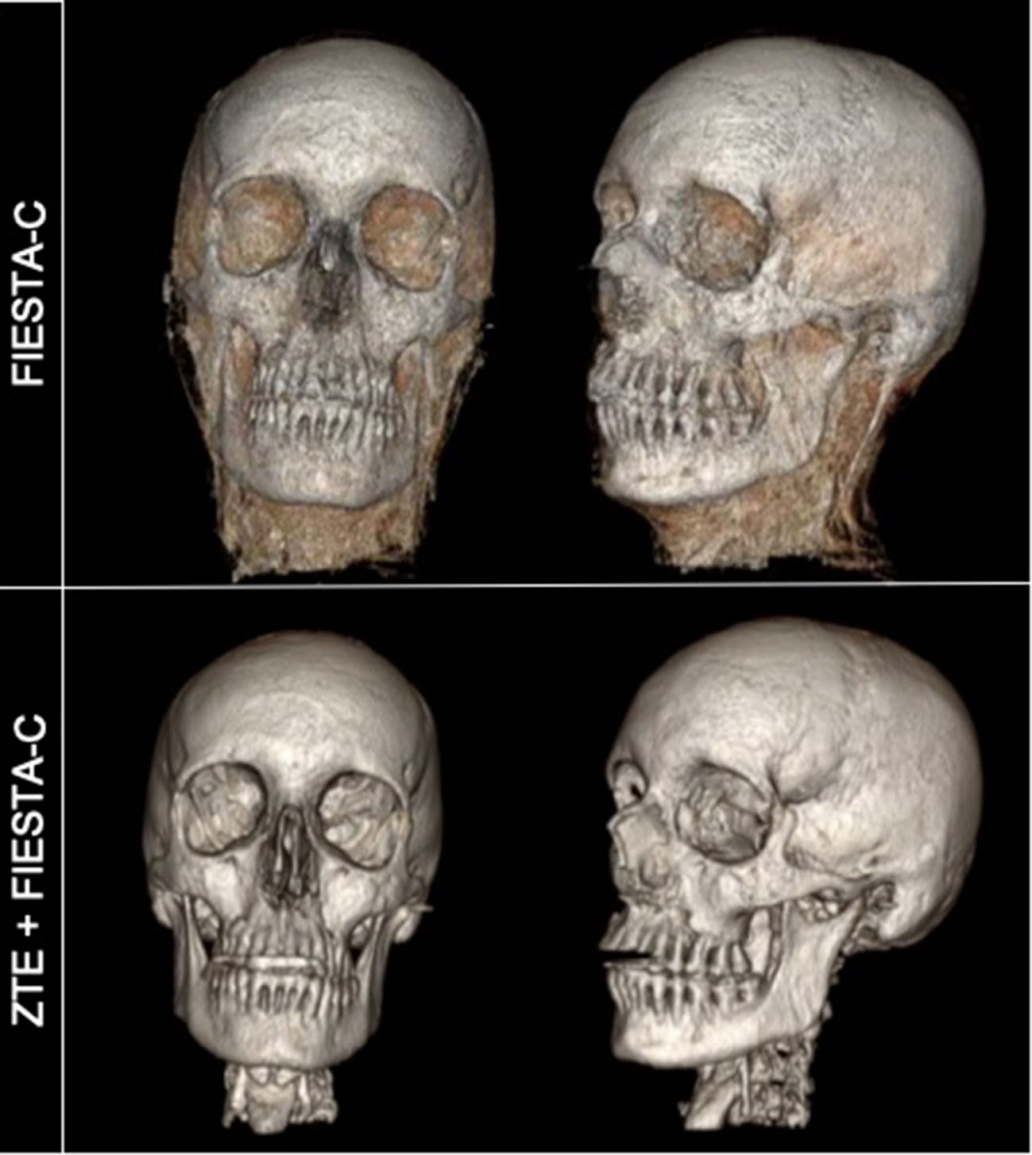
Automated 3D reconstruction of the craniofacial skeleton in adult volunteers using FIESTA-C and a modified algorithm with ZTE+FIESTA-C

The combined acquisition time for FIESTA-C+ZTE was 4:44 min. The standard and modified algorithms were both transferrable to FIESTA-C datasets, which demonstrated comparable 3D outputs to BB (Fig. 7).

The image processing time to produce the automated 3D outputs was on average 9 min 30 s per dataset on a standard Intel i7-9750H 2.60GHz CPU laptop, without any code optimisations (average CPU usage approximately 10%).

## Discussion

We have demonstrated that automated segmentation of the craniofacial skeleton is enhanced using a modified algorithm using ZTE to drive the segmentation process. This takes advantage of the integral benefits of BB and ZTE; BB offering high-resolution imaging in a short time, and ZTE discerning bone from air and unwanted tissues. As such, ZTE identifies where bone is, and BB is used to segment bone in those regions. There are two main advantages to this modified technique; firstly, there is improved separation of bone from confounding structures such as tendons, saturated long-T1 structures, tissue interfaces and intensity inhomogeneities, and secondly, and perhaps most importantly, the ability to accurately identify air. Sites where air and bone are in close contact have previously been the fundamental limitation of 3D BB techniques, since the identical intensity of bone and air resulted in an inability to separate these two entities. The ability to now distinguish air and bone opens up a wide range of potential applications bringing 3D reconstruction closer into the realm of becoming a viable alternative to CT. It should be noted that air located within the oropharynx and paranasal sinuses were not specifically targeted for segmentation in this initial study, and are thus visualised in the 3D reconstructed images. These air-filled structures, affected by partial volume, were not accurately identified due to a residual layer of incorrectly defined bone, inaccuracies which could be resolved using more sophisticated processing such as DL-based techniques. These could be easily removed manually by the end-user in software such as Osirix.

A further area of discrepancy occurred as the result of leakage during masking, which resulted in missing slices of data at the teeth with a resultant absence of the expected anatomical convolutions at the occlusal plane. This is deemed a minor trade-off for improved segmentation of the craniofacial skeleton.

Improved segmentation of the nose was seen with the modified algorithm as a result of improved distinction between the cartilage and nasal bones. This area was previously particularly challenging to segment on BB datasets, requiring manual removal. However, there remain some areas of inaccuracy at this sub-site as a result of post-processing and as such are amenable to further improvement in the future.

Post-processing errors also resulted in artificial areas of discrepancy occurring in regions such as the skull vertex, highlighted through review of the raw processed datasets and subsequent volume rendering of this data in Osirix. This was likely due to a combination of mis-registration between ZTE and BB, as well as mismatch of the voxel size in z direction, and post-processing error. This could be addressed with more isotropic voxels, image registration prior to processing and subsequent iterations of the 3D processing pathway.

The techniques used to produce the 3D outputs in this study maintain imaging information in the processed dataset to permit the end-user to enhance the final 3D reconstructed image that is displayed using software such as Osirix. In essence, segmentation of images into bone masks requires a binary distinction, bone or not-bone, which can be difficult in some anatomical regions. This would remove all possibility of manipulating the final image, since anything deemed “not-bone” in the processing phase would be completely lost. Our approach was to remove only structures that we are certain are of no clinical interest, but preserve intensity information so that the user can utilise their knowledge of anatomy and experience to manipulate the rendering and get the best possible 3D image—this is where the advantageous contrast of BB data is exploited. However, this does mean that it is very difficult to perform any quantitative comparison between 3D images created in this way. This is the main limitation of the study, and unfortunately none of our volunteers had prior CT imaging to compare their 3D results with. Whilst our ongoing patient recruitment will provide us with comparable CT imaging (acquired for clinical indications), quantitative comparisons will not be possible due to the lack of fully segmented bone masks, a trade-off to provide enhanced 3D imaging. Whilst we chose to undertake manipulation of the final 3D images in Osirix in this study, since the post-processed segmented data is exported in DICOM format, this could be performed using any 3D rendering software of the user’s choice.

The short acquisition time of just over 1 min for ZTE minimises any potential mis-registration of the BB and ZTE datasets due to patient movement between the two acquisitions, but this remains a potential limitation of the technique. The combined BB-ZTE acquisition time of 6:04 min, whilst comparable with many routinely used sequences, is not ideal in the subset of patients most likely to benefit from these techniques—young children. Young patients are most prone to motion artefact without the use of sedation or general anaesthesia within the MRI environment, both of which are not risk-free. Methods to further reduce acquisition time for bone imaging continue to be explored. FIESTA-C offers a slight advantage over BB in this regard, with an acquisition time approximately 1 min shorter, with a combined FIESTA-C+ZTE acquisition time of 4:44 min whilst still achieving comparable 3D results. The acquisition times in this study overall compare favourably with the majority of the prior studies reported in the literature which have focused on dual-echo UTE, or ZTE imaging techniques. In the study by Kramer et al. [1], the dual-echo UTE acquisition protocols were “as short as 6 minutes depending upon the spatial resolution, with high-resolution protocols taking significantly longer.” In the 8 adult volunteers, the reconstructed 3D surface renderings showed a smooth segmentation of the cranial bone without holes or other superficial artefacts, but suboptimal reconstructions of the facial skeleton were achieved. Wiesinger et al. [13] focused on ZTE with a scan time of 6 min 12 s required to acquire the high-resolution imaging necessary to create 3D reconstructed imaging. Lu et al. [11] reported improved scan times of 4–5 min by using two separate ZTE acquisitions per patient. In their study, they imaged 14 patients aged between 3 months and 17 years with various pathologies, but provided only one example of the representative 3D output which appeared to be from an adult (no data provided). It is therefore not possible to make comparisons between the successes of the 3D outputs. It should also be noted that a number of the 3D reconstructions included in the published literature have utilised a high smoothing factor which enhances aesthetics at the cost of reduced accuracy.

The image processing time to achieve the automated 3D outputs in our study was relatively long (average 9mins 30 s per dataset). The primary focus of this initial study was on achieving the most optimised 3D imaging possible. Code optimisation in the future would have a significant impact on these processing times, with an expectation that this could be reduced to less than 2 min and possibly even further if completed directly on the MRI console. It should be noted that since no user interaction is required, post-processing could be performed in parallel with acquisition of any additional sequences in the MRI examination, thus having minimal impact in the clinical environment.

Utilising ZTE in combination with a sequence which offers superior imaging resolution overcomes the inherent limitation of ZTE. The ability to select FIESTA-C or BB depending upon the clinical indication also widens the potential application for these techniques. For example, bone marrow signal, particularly within the mandible, is more uniform on BB than FIESTA-C which would lend itself to improved visualisation of tumour infiltration, whilst FIESTA-C imaging offers enhanced visualisation of the cranial nerves at the skull base and high T2 imaging of the membranous labyrinth. FIESTA-C imaging is however more susceptible to pulsation effects than BB.

There is no shortage of potential applications for 3D reconstructed imaging and 3D models of the cranium and facial skeleton, with ongoing research into MRI methods for neuroscience applications and surgical planning [14–17]. The field of craniomaxillofacial surgery remains a key driver in this quest in view of the multiple congenital and acquired conditions requiring frequently challenging (and often multiple) surgical interventions. Non-accidental injury in the paediatric population offers similar challenges with a need for comprehensive and ideally non-ionising imaging. The potential for BB and the ZTE equivalent, PETRA, techniques in the diagnosis of craniosynostosis and skull fractures has been demonstrated [3, 8]. The demonstrated benefits of a combined ZTE+BB or ZTE+FIESTA-C segmentation algorithm would be of clear benefit in these patient groups, albeit with the requirement for further confirmation of their accuracy. We continue to explore these methodologies with ongoing refinements in a larger study focused on infants with craniofacial anomalies in whom the potential benefits would be perhaps greatest, and in whom comparable CT imaging provides an ethically sound comparator.

In conclusion, automated 3D reconstructed imaging of the craniofacial skeleton is enhanced through the incorporation of a modified segmentation algorithm using ZTE. These techniques are transferrable to FIESTA-C imaging, which offers improved clinical utility.

## Abbreviations

3D: Three dimensional
BB: Black bone
GRE: Gradient echo
GRE-BB: Gradient echo black bone
UTE: Ultra short echo time
ZTE: Zero echo time
